# Learning sites for health systems research: Reflections on five programs in Africa, Asia, and Central America

**DOI:** 10.1002/lrh2.10475

**Published:** 2024-12-04

**Authors:** Sophie Witter, Shophika Regmi, Joanna Raven, Jacinta Nzinga, Maria van der Merwe, Walter Flores, Lucia D'Ambruoso

**Affiliations:** ^1^ Institute for Global Health and Development Queen Margaret University Edinburgh UK; ^2^ HERD International Kathmandu Nepal; ^3^ Liverpool School of Tropical Medicine Liverpool UK; ^4^ Health Economics Research Unit, KEMRI Welcome Trust Research Programme, Nairobi, Kenya & Liverpool School of Tropical Medicine Liverpool UK; ^5^ Independent Consultant White River South Africa; ^6^ School of International Service American University Washington DC USA; ^7^ Centro de Estudios para la Equidad y Gobernanza de los Sistemas de Salud Guatemala City Guatemala; ^8^ Centre for Health Data Science, School of Medicine, Medical Sciences and Nutrition, and Centre for Global Development, School of Education University of Aberdeen Aberdeen UK; ^9^ MRC/Wits Rural Public Health and Health Transitions Research Unit (Agincourt), School of Public Health University of the Witwatersrand Johannesburg Johannesburg South Africa; ^10^ Department of Epidemiology and Global Health Umeå University Umeå Sweden; ^11^ Public Health, National Health Service (NHS) Grampian Aberdeen UK; ^12^ Department of Global Health Stellenbosch University Stellenbosch South Africa

**Keywords:** health policy and systems research, learning sites, low‐ and middle‐income countries, participatory action research

## Abstract

**Introduction:**

Learning sites have supported intervention development and testing in health care, but studies reflecting on lessons relating to their deployment for health policy and system research (HPSR) in low‐ and middle‐income settings are limited.

**Methods:**

This experience report draws from learning over three continents and five research and community engagement programs—the oldest starting in 2010—to reflect on the challenges and benefits of doing embedded HPSR in learning sites, and how those have been managed. Its objective is to generate better understanding of their potential and constraints. The report draws from team members' experiential insights and program publications.

**Results:**

Challenges relating to initial engagement in the sites included building and maintaining trust, managing partner expectations, and negotiating priority topics and stakeholders. Once the embedded research was underway, sustaining engagement, and managing power dynamics within the group, supporting all participants in developing new skills and managing rapidly changing settings were important. Finally, the complexity of reflecting on action and assessing impact are outlined, along with potential approaches to managing all of these challenges and the variety of gains that have been noted across the programs.

**Conclusions:**

We highlight the potential of learning sites to develop relationships, capacities, and local innovations which can strengthen health systems in the long term and some lessons in relation to how to do that, including the importance of stable, long‐term funding as well as developing and recognizing facilitation skills among researchers. Supporting spaces for learning is particularly important when health systems face resource constraints and everyday or acute stressors and shocks.

## 1 INTRODUCTION

Learning sites are research platforms established in a specific geographical area, where researchers and local actors collaborate over an extended period of time to develop contextually tailored interventions.^1^ The type of research conducted and the actors involved will depend on the focus of the learning sites, but typically, in HPSR, the process of enquiry is emergent within a broad framework, with a commitment to joint ownership and co‐production of knowledge (Box 1).

BOX 1 **Typical features of learning sites.**

1. They are based on long‐term collaboration between researchers and policy‐makers and/or communities—building trust through sustained engagement.
2. They provide a platform for action research—knowledge for local use.
3. They focus on co‐production of knowledge, recognizing the importance of local lived experiences, experiential knowledge, tacit knowledge about health systems and decision making, and local relationships.
4. They recognize and attempt to rebalance power dynamics in the program (between international and national, but also community and system actors, and sometimes researchers versus implementers).
5. Researchers are often embedded in or interacting regularly with the health system; through research activities, they come to understand the daily routines and challenges faced by health managers and other actors.

Source: (Gilson et al.,^1^); authors.

However, while potentially a very powerful strategy, such an approach is not without ethical, practical and other challenges. For example, Oliver et al. highlight risks of co‐production relating to partiality (e.g., where researchers only report what is judged acceptable to policy or management partners).^2^ Learning sites also need to manage power relationships, working politically as well as technically, and managing system constraints (including limited resources) in relation to implementation and research. Sustainability, institutionalization, and scale‐up are also typically areas of challenge, given the intensity of effort devoted to relationship building in many learning sites.

Although this approach has been adopted by a number of research programs, there is relatively little published reflection on health system learning sites in low‐ and middle‐income settings, how they function, what their strengths are, what challenges they face, and how to overcome them. In this paper, we draw together reflections from five research programs which have employed methods that fit within a broad learning site approach. We reflect on emerging lessons, considering the different models that have been adopted, the diverse objectives and varied settings in which they have been employed (in programs based in three continents), as well as the complex and long‐term processes of health system improvement and change.

The paper was developed following a panel session on this topic in the Global Symposium on Health Systems Research 2022 and builds on reflections within and across research teams, as well as the participant questions and feedback which were shared during the panel discussion.

## 2 BACKGROUND ON THE PROGRAMS

Table 1 gives a summary overview of the five programs, which were conducted in sites in Nepal, South Africa, Kenya, Ghana, Uganda, Malawi, and Guatemala. The size of sites varied, and most were rural, with the exception of ReSYST in South Africa. Two of the programs are completed (PERFORM2Scale and ReSYST), one is ongoing but time‐limited (ReBUILD), one is currently being scaled‐up (VAPAR), and one is a long‐term research center, with a variety of funding sources (CEGSS).

**TABLE 1:** Summary of five research programs using learning site approaches.

Program	Objectives	Funder and dates	Setting and coverage	Focal topic and approach
ReBUILD for Resilience (www.rebuild.consortium.com)	To provide contextualized evidence on how to develop resilience capacities for health systems in fragile and shock‐prone settings	FCDO 2020–2026	Learning sites have been developed in a municipality of Kapilvastu district, Nepal; also in the Bekaa valley, Lebanon; a similar approach is being deployed in Kailahun and Moyamba districts in Sierra Leone. This paper focuses on the Nepal experience, as this was the first to be initiated. Kapilvastu is a semi‐urban district in Lumbini province, in the lowland (*terai*) area of Nepal, bordering India. The municipality is fragile in many ways due to its poor health service indicators compared to national average, weaker health infrastructure and access to routine health services and cross‐border migration, which increases vulnerability to disease and infection. The main source of income is agriculture, and lack of opportunities for work have increased labor migration. The population of the learning site is around 77 000.	Since federalization in 2017, planning and delivery of health care has been devolved to the municipality level in Nepal; however, capacities at this level remain to be built and systems (and connectors) in this new arrangement need to be established. An embedded approach of implementation research involving a researcher stationed within the municipality and working with local health systems stakeholders has been adopted. The action research steps are ongoing (12–18 months) and include: Partnership development Participatory mapping of resilience capacities Co‐creation of interventions to strengthen resilience capacities Evaluation of implementation and the PAR cycle Although the resilience mapping is broad (including most health system blocks), key areas emerging to date have been governance and use of local data for planning.
PERFORM2Scale www.perform2scale.org	To strengthen district level management to address workforce performance and service delivery challenges	EC (Horizon 2020) 2017–2022	PERFORM2Scale program worked in learning sites in Ghana, Malawi, and Uganda. In each country, we established three learning sites which were groups of three district health management teams that were in neighboring districts. Willingness to participate, established relationships, and avoiding districts where similar interventions are being implemented were important criteria for selection. The districts were mainly rural but some with a city or large town. Populations of the districts varied, with Ghana's districts being smaller at around 100 000, whereas in Uganda populations they were between 300,00–500 000, and in Malawi, populations ranged from 200 000 to 1 million.	The management strengthening intervention (MSI) used a PAR approach to enable the district health management teams to analyze their own problems related to workforce performance and service delivery and develop appropriate work plans (plan); implement the work plans (act) and learn about management from the experience (observe and reflect). The MSI was facilitated by national research teams and government officials in each country through short workshops, joint meetings of DHMTs, and follow‐up support by visits, e‐mail, and telephone/Whatsapp. The cycle took about 10 months and then moved into another cycle, either adapting the strategies to address the same problem, or addressing another problem.
ReSYST https://resyst.lshtm.ac.uk/	To examine health system routine stresses and everyday resilience, including how health managers' leadership practices, organizational relationships and their underpinning values influence health system resilience	DFID (now FCDO) 2011–2018	Two sites were developed: Kilifi County in Kenya, and in South Africa, two health districts located in different provinces (Sedibeng, Gauteng; and the Mitchell's Plain area of Cape Town, Western Cape). These sites were initially chosen because of prior experience and engagement with managers. Research teams live locally and have maintained trusting relationships with health system colleagues over time. Kilifi is a rural setting compared to the urban contexts of the South African sites, and Mitchell's Plain is a sub‐district rather than a district, with a population (around 500 000 people) about half that of the other sites. In Kilifi, the population (1.5 m) is relatively poor. Research teams live locally and have maintained trusting relationships with health system colleagues over time.	ReSYST focused on leadership practices and organizational relationships, with the aim of building and supporting soft skills (such as communication, motivation and responsibility) among leaders and at multiple levels of the county health system (e.g., through coaching initiatives and participatory training in communication with colleagues and emotional management). Core approaches were collective enquiry and reflective practice. More specifically, in selected facilities, it tracked and investigated specific chronic stressors (e.g., absenteeism, patient complaints) and potential alleviators of chronic stress (e.g., new public finance policies or supervision approaches and guidelines) identified with mid‐level and facility managers as possibly having important implications for system resilience. Beyond tracking the stressors, it continued tracking implementation and impact of past initiatives (South African sites), and development and tracking of new initiatives (all sites) aimed at strengthening health system governance. Some of these initiatives included coaching interventions at senior & facility management levels (implemented in previous phase of learning site work) and adaptation of a soft skills communication and emotional management training course for the managers.
CEGSS (Centro de Estudios para la Equidad y Gobernanza de los Sistemas de Salud) https://cegss.org.gt/en/	To demonstrate that participatory governance of municipal healthcare facilities is feasible and brings better results. In the sites, the program also develops and field tests methods and tools for citizen monitoring and authority responsiveness.	Internationally funded through private foundations, universities and research institutes 2010‐present	CEGSS is present in 35 municipalities, working together with the Network of Community Health Defenders (REDC‐SALUD), which are over 150 organized community leaders. However, not all municipalities are demonstration sites. Demonstration sites are chosen where there is combination of authorities' openness, grassroots capacities, and channels of engagement between users of services and authorities. There are currently 8 in areas with diverse geography and ethnic population groups. In other sites, CEGSS works to generate or strengthen these conditions. Municipalities vary in population size (between 5 K and 15 K inhabitants). However, the focus of the demonstration sites is on the network of public healthcare facilities in the territory. On average, there are one or two health centers per site and several health posts, which are the most peripheral healthcare facilities.	Demonstration sites are municipalities in rural indigenous areas in which CEGSS, together with grassroots organizations, reflect, design, implement, evaluate and learn about strategies for citizen‐led health accountability. These processes are based on PAR cycles. In all sites, there is collaboration with providers and authorities. However, in all places the process is led by organized users of services. The approach is based on longstanding mentoring of community leaders, whose families and neighbors use public services. The purpose of the sites is to generate learning on effective strategies and capacities to improve democratic governance of the health system. The learning sites also host visits from grassroots organizations, providers, and health authorities. These visits motivate other actors to replicate the approach and adapt strategies and methods to their own context. In addition, the reflection and learning among all sites – including those that are not demonstrative – is shared and disseminated through the REDC‐SALUD. The goal for CEGSS and REDC‐SALUD is that through action research, community organizing, and capacity building, all the 35 municipalities will eventually achieve the favorable conditions of the learning sites.
VAPAR https://www.vapar.org/	To develop inclusive knowledge partnerships to strengthen health systems; evaluate changes in health, health equity, and empowerment; and build sustainability and transferability	Medical Research Council/HSRI (with additional funds from the Newton Fund, GCRF and Scottish Funding Council) Development grant: 2015–2016; main grant: 2017–2023	VAPAR was based at the MRC Wits/Agincourt Health and Socio‐Demographic Surveillance System (HDSS) in Bushbuckridge sub‐district, Mpumalanga, South Africa. In the HDSS area (population 120 000), rural homesteads experience multigenerational deprivation. There is little formal sanitation, unaffordable electricity, high unemployment, and a limited economic base resulting in labor migration and reliance on social grants. Orphaned youth characterize the population: school drop‐out is 40%, 16% of the provincial population is illiterate, and district unemployment is 37%. The burden of disease is complex and dynamic: while HIV/AIDS and maternal mortality and child deaths are decreasing, non‐communicable conditions, including deaths owing to injuries, accidents and violence, and health disease, are increasing, together with chronic, complex comorbidities in an aging population. There are two community health centers and seven PHC clinics.	VAPAR supported a series of reflection/action cycles to generate information on collectively identified health concerns in terms of associated disease burdens, their social and health systems determinants (using VA), and on norms, practices and priorities for action from the perspectives of directly affected and under‐represented groups (using PAR). Local data were developed together with community capabilities for these data. Data were then collectively analyzed in a multi‐level process of health systems engagement including with DHMT, through which recommendations, local action plans, implementation, evaluation, reflection and adaption were co‐developed, and which fed learning into the next cycle. Co‐produced evidence was fed into action in the local health system. Themes were initially nominated by communities as alcohol and drugs, and water security (Cycles 1–2). Later, cycles (Cycles 3–5) prioritized dialogue with local health systems actors and focused on CHW capabilities, roles, and functions; specifically, HIV/TB treatment. CHWs reported improved relationships with communities, peer support, and recognition by the health system. The process is currently being adapted for scale‐up from the rural sub‐district (population approx. 500 000) to the province (population 4.8 million).

In terms of focus, three were focused on the health system (supply‐side), while two (VAPAR and CEGSS) had a stronger community‐led approach, albeit also engaging with health system strengthening. Two took resilience as their core theme—one in more stable contexts (ReSYST) and one in fragile settings (ReBUILD), while PERFORM2Scale focused on strengthening management to address workforce performance and service delivery challenges and CEGSS on participatory governance and social accountability. VAPAR has focused on more specific health topics, nominated by community stakeholders in collaboration with health systems actors at district, sub‐district, clinic and community levels, which framed community‐nominated priorities in terms of their social determinants, thereby connecting the process with sectors adjacent to health.

All used participatory action research (PAR) methods, seeking leverage points for change and focused on positive system and community assets and capabilities, and using the cycle of collective analysis, co‐creation of responsive and evidence‐based plans, their implementation, and reflection and feedback on action as an active intervention in its own right.^3^ All programs had additional elements, such as coaching, mentoring and training (all five programs). In addition, VAPAR drew on verbal autopsy (VA) data generated by the Health and Demographic Surveillance Site (HDSS) in which it is based to support participants in planning and priority setting with robust local data on burden of disease. VAPAR also extended the VA method to collect and interpret data accounting for social and health systems determinants of mortality.

Research methods used were/are primarily qualitative and participatory, including document review, individual and group interviews, critical reflection, use of diaries and case studies, observation, reflective meetings, participatory workshops (including group model building), and photovoice.

## 3 CHARACTERISTIC ISSUES FACED BY LEARNING SITES AND HOW THESE WERE MANAGED

A number of challenges were experienced across the learning sites, across the different stages of the learning process. We highlight these here with some examples of how they were managed by the programs and some lessons learned (see summary in Table 2).

**TABLE 2:** Challenges, how they were managed and lessons learned.

Challenges encountered	How these were managed (examples of strategies)	Lessons learned
Initial engagement		
Building and maintaining trust	Developing a shared vision of purpose and process, and adapting this collectively to changing needs and circumstances Allowing time for relationships to develop Progressively shifting control to participants Capacity building of participants (communities, health system actors, and researchers) Using local data to show utility of work and engagement with local context Working flexibly around participant constraints Demonstrating value through effectiveness in engagements Bringing in other actors when appropriate and requested	Trust is gained and retained by continuous enactment of respectful and responsive contributions of the members of the platform, while acknowledging positionality and limitations Important to help forge networks for participants, both horizontally (linking with other areas to share and support) and vertically (given that many issues can only be addressed effectively at higher levels). Systems tend to work in silos; learning platforms aim to break these silos
Managing expectations (of additional resources)	Being transparent about roles Linking partners to wider resources and building their skills to be able to access them	
Priority‐setting dilemmas	Enabling/empowering participants to select and frame local priority health concerns Building on these to identify and progress shared agendas with authorities In some cases, prescribing focal areas which linked to sectoral interests	
Choosing level of engagement	Working at multiple levels of the health and wider system to ensure that systemic factors can be addressed Building confidence of local stakeholders to address priority issues, despite limited decision space Building networks and sharing lessons across them	
Managing the process		
Maintaining commitment from participants and funders	Demonstrating agility and adaptability to respond to local needs, including by changing focus during crises Showing wider utility by providing support to local priority activities not included in program plans Building collective capabilities and mindsets Including local leaders as participants and co‐authors Using formal tools such as MoUs to institutionalize engagement, even as local leaders change Using local platforms and aligning to planning and budget cycles Effectively including community members, supporting a rights‐orientation Extending engagement to a wide network (planning for expected attrition over time) Demonstrating collective effectiveness Critically engaging in academic and funder debates on value of PAR Creating supportive environment for researchers (e.g., capacity development and equitable publications)	In constrained environments, pragmatic use of existing structures and fora is important to encourage engagement and sustainability We also noted the importance of working multisectorally, especially in decentralized environments where health is not necessarily a priority for local leaders. Empowerment by learning and doing is key
Managing power dynamics within group (to ensure participation of marginalized voices)	Sensitive but assertive facilitation Jointly establishing values of democratic participation, voice, and mutual respect and regularly reinforcing these Separating groups where appropriate Acknowledging power differentials, also for and within researcher group Acknowledging systemic challenges, even when these are sensitive	Facilitation skills involve a complex mix of technical and inter‐personal skills; these can be developed over time but need recognition and nurturing Creating a collective and problem‐solving mindset can help to overcome differences of positionality and power
Developing new skills for researchers and participants	Training and practice in PAR, data analysis and writing for participants (democratizing research methods and adapting them for use by those dealing with the burden of disease) and in planning and supporting implementation for researchers Embedded approaches—learning by working alongside one another, gaining better mutual understanding of opportunities and constraints	Spaces for dialogue that link different groups and resources are often missing; by creating these, opportunities for learning are created
Reflection on action		
Complexity of monitoring, reflection and learning	Avoiding additional workload by building reflection into existing fora Holding regular reflection and sensemaking meetings, with systematic and transparent recording Establishing virtual platforms and shared repositories as useful tools	Continuous critical reflection is needed to ensure that all voices are heard, collectively validated and acted on Engaging wider networks to reflect on lessons learned in one area can enhance relevance of learning for wider geographies
Assessing impact	Theory‐based evaluative approaches can help assemble evidence on the complex changes sought and (variably) achieved, using mixed methods PAR generates data on a continuous basis, which can be used for cycle evaluations, as well as regular reflections Capacity assessment is complex but can be addressed using multiple qualitative methods, for example, observations, interviews, and researcher logs	While valuing health and health equity gains, it is important not to overlook the importance of “intermediate” stages in the theory of change—such as strengthened relationships and demand for evidence—especially since these shape the sustainability of the learning platform and its ability to support future and wider benefits Learning sites produce intrinsic as well as instrumental benefits, but conventional evaluation metrics tend to neglect or underplay the intrinsic

### 3.1 Initial engagement

#### 3.1.1 Building and maintaining trust

Trust was highlighted as crucial to developing the learning site relationships. Approaches to earning trust included regular revisiting of PAR principles to support shared vision and purpose and allowing time for relationships to deepen. For example, in VAPAR “engagement gradually improved as core principles were transmitted, discussed, revisited, owned, and taken up. Ownership was supported as participants assumed control of the process: identifying priority health concerns, directing expansion of the participant base, and controlling practical aspects such as dates, times, and venues of workshops.”^4^

The importance of positive relationships was also highlighted in ReSYST—ensuring that participant ideas are incorporated, that feedback is regular, and that activities are flexible and agile to respond to heavily politicized contexts, demanding job environments and frequent turnover of post‐holders. Facing and ultimately overcoming challenges also served to build mutual understanding and trust in all settings.^5^

Capacity building through training and mentoring also developed trust across programs. In VAPAR, collective capabilities, mutual understanding, and trust developed as participants' familiarity were built with public speaking, analysis (including mapping causes and consequences of local health priorities, and in selecting, appraising, and captioning visual data), consensus building, peer and non‐peer deliberations, and in co‐facilitation and recording of meetings.^6^

In Nepal, the use of local data and their intersectional analysis proved to be a key instrument in building trust and establishing relationships with local counterparts. It supported showing the strengths and capabilities of the research team, which the local government, transitioning to federalization, acknowledged and in response sought technical support to strengthen their health system.^7^

#### 3.1.2 Managing expectations

Particularly at initiation but also on an ongoing basis, managing participant expectations is challenging, particularly in contexts, such as Nepal's, where financial dependence on external development partners is high and there is a perception that resource limitations are the main obstacles to improving health services.

This can be managed through continuous and frequent engagement, being transparent about the approach and facilitating role of the research team, helping to connect partners to wider resources but also helping them to identify what can be done more effectively within existing means.^8^ Over time, partners have come to see the high value of the role played by the learning platform and have adopted entrepreneurial approaches to resourcing planned activities.^9^

#### 3.1.3 Priority‐setting dilemmas

The challenge of prioritizing focal problems to address arose for some of the programs. For example, in VAPAR there were some initial challenges as community‐nominated topics in the first two cycles such as access to water were seen as less tractable by health system actors. However, the district health system actors appreciated the rare opportunity to connect cross‐sectorally.^10^, ^11^ Latterly, in response to adaptation during COVD‐19, cycles focused on the roles and functions of CHWs. This worked well as this group connects communities to the health system and so engaged the key points of the community, system, and researcher triangle with a shared priority (addressing loss to follow up for TB and HIV treatment).^4^, ^6^ Other programs resolved the challenge by defining clear areas of engagement—for example, PERFORM2Scale focused on workforce performance and service delivery, but within that each district chose the problem that they wanted to address. This fostered more engagement in finding solutions and implementing them. District managers were used to development partners and others identifying the problems that should be addressed.

#### 3.1.4 Choosing level of engagement

Assessing whether to stay focused or work at multiple levels of the system is one of the choices facing learning sites. All programs worked at multiple levels of the health system, recognizing that change involves mobilizing stakeholders with different roles in the health system and at community level. However, getting traction at higher levels can be challenging. In Guatemala, for example, the demonstration site work can be seen as threatening by national authorities. The sites encourage an active role by citizens which can threaten the power of officials. However, some authorities and elected officials are supportive of the work all the same.

Engaging those with some degree of power to act was critical. Some programs addressed this by working at multiple levels of the health system (e.g., in ReSYST), while others engaged a wide range of actors from beyond the health sector (such as local government in ReBUILD and PERFORM2Scale and multiple sectors in VAPAR). However, structural and political issues constraining decision space of local managers are typically hard to challenge.^12^ The programs noted the importance of building confidence of managers to be able to analyze, strategize, and implement plans for improvements and to be able to articulate these improvements and advocate to higher levels of the health system and other sectors for financial and other support, working in their own teams but also sharing lessons across sites (which can also encourage co‐learning and exchange).

### 3.2 Managing the process

#### 3.2.1 Sustaining engagement

Maintaining continuity of commitment, including from partners and funders, is a challenge across all learning sites. For partners, it is important to demonstrate the utility of the approach, while keeping expectations grounded. Many programs have responded adaptively to acute and chronic crises, providing additional support during COVID‐19, for example. One approach adopted in Nepal was to demonstrate commitment to local government by supporting them not only in activities outlined in the action plan but also in their day‐to‐day programmatic and administrative tasks. This significantly contributed to their capacity enhancement and strategic planning and execution.

More profoundly, it is fundamental that collective mindsets and capabilities for joint action and learning are built, which can sustain the engagement over the longer term. In VAPAR over five reiterative action/learning cycles, the local action plans became more specific, strategic, focused, and feasible, reflecting capabilities built through sustained engagement in the process. Local leaders at multiple levels were included as partners from the start of the program and were encouraged to participate in all stages of the cycles, including as co‐authors of outputs and publications, but also in training and program development.

In dynamic contexts, such as the decentralized health system in Nepal, changing office‐holders and uncertainty of roles creates additional operating challenges for learning sites, given their long‐term nature. A memorandum of understanding to agree on roles, regardless of changes in leadership, as well as participating in and contributing to routine municipality meetings and workshops is how the research partners (HERD International) worked to maintain relationships in this context. Maintaining multiple relationships and being adaptive to changes is however resource‐intensive, so budget sufficiency and flexibility is needed. For example, the research teams need to align the research pace with the interests and priorities of local counterparts, particularly in the initial phase of the project. They cannot impose planned research activities over their priorities, which leads to delays. It took 1 year for the Nepal team to co‐create the action plan (following resilience mapping and participatory workshops). Countrywide elections, local planning, and several other factors affected the process.^7^ Equally, COVID required a pivot in focus and activities for programs such as VAPAR and PERFORM2Scale, where managers applied problem analysis and solving to addressing COVID‐19 in their districts.^13^

For community members, feeling recognized, respected, and valued in their contributions, as well as seeing responses in terms of action and follow‐up, are key to continued engagement. Changing mindsets is key here too—citizens need to come to view health care as a right and believe that they can claim better services, as highlighted in the CEGSS model. In addition, recognizing that community activists are volunteers, who may reduce their participation and engagement at different times, additional training is done by CEGSS with communities to allow for replacements. The number of community defenders (as the activists are called) has increased by 40% in the past few years, which contributes to sustaining the work.

According to our experience in learning sites, researcher engagement also needs to be sustained, as academic environments do not typically support, enable, or reward embedded and PAR approaches, which are seen as delivering very contextual and “low grade” evidence, with considerable energy devoted to local relevance and uptake (more than high‐ranking publications). Funding does not typically make provisions for stakeholder/policy engagement activities and particularly for the substantial periods that are needed to demonstrate impact. In part, this is addressed by selection of researchers with a particular orientation to applied research, but also through providing a supportive environment in terms of training opportunities and support for equitable authorship and networking. It is also important to engage in critical debate in academic spaces to highlight the value and rigor of enquiry paradigms concerning knowledge for action, plurality of knowledge, cooperative learning, and expertise from the margins. It is also important to engage funders on the value of learning site approaches and to sustain their support as long‐term engagement is a key requirement for learning site effectiveness.

#### 3.2.2 Managing power dynamics within group

All programs aimed to include marginalized voices, but especially so for those which focused at community level, such as CEGSS and VAPAR. Including affected people and those whose voices were excluded required very careful management of group dynamics to ensure inclusion, participation, and respectful engagement. In VAPAR, it was beneficial to start by spending significant time building community capabilities for community voice, together with local, actionable data, as the basis of engagement with the authorities.

During workshops with representatives of the authorities, some stakeholders were dominant and disruptive, leading to others feeling intimidated to raise their opinions. In some instances, VAPAR observed local politicians using the platform to promote current priorities or debates. It dealt with this with sensitive, but assertive, facilitation, reinforcing principles of democratic participation, voice, representation, and respect. Over time, the regular negotiating of these principles supported mutual understanding, supported agency and more equal participation. Facilitation skills of the engagement process were also very key in all programs, blending the technical knowledge and expertise of the research team and the art and skills of facilitation. Separate workshops with different sets of stakeholders were another strategy to manage power dynamics and enable stakeholders to express themselves (e.g., in Nepal, health workers and elected official met separately due to the nature of their relationship with one another).

Power imbalances also relate to researchers, who potentially have different access to opportunities and resources within their network, compared to communities and system actors. This has the potential to undermine the relationship, if not acknowledged and addressed, in our experience. Highlighting systemic challenges can also be uncomfortable for local stakeholders, and researchers need to be able to address such topics in a sensitive but open way.

#### 3.2.3 Developing new skills for participants and researchers

In learning sites, researchers actively facilitate and support partners. This is a marked departure from the conventional positivist role of researchers, which is to objectively study phenomena free of subjective influence or “contamination.” This was challenging and involved capacity building for researchers and partners, who are also being supported to develop a more problem‐solving and analytical mentality. The research team performed the dual roles of researchers and implementation support practitioners, which required knowledge and skillsets, as well as a deep understanding of the context, stakeholders, and power relations. Without this type of implementation software work, progress with implementation is challenging.

These new skills are supported by learning through doing, but also through training, mentorship, and the development of supportive tools and toolkits (e.g., PERFORM2Scale developed a toolkit for facilitation [^14^], while VAPAR developed a community mobilization toolkit in collaboration with the Department of Health for all participants)^15^ and a post‐graduate health policy and systems research module to support wider application of methods and tools.

Strengthening capacity and institutionalizing learning are the core principles of engagement in the learning sites. Research partners providing technical support, such as HERD International in ReBUILD, have been careful to transfer skills and knowledge (e.g., in developing evidence‐based local plans), focusing on institutionalization of practices to avoid dependency. Equally, where capacity gaps become evident, research partners have been able to respond flexibly to support filling these, given their embedded position (e.g., in Mpumalanga, VAPAR supported the capacity building and development of research governance resources and training with the provincial research ethics committee, which had not been planned originally).^16^ In ReSYST, discussion of the learning site concept and sharing of literature, narratives, and ideas were used to increase participant confidence and support progress with analysis and writing up of learning.

### 3.3 Reflection on action

#### 3.3.1 Complexity of reflection, monitoring, and learning

All programs built reflection, monitoring, learning, and evaluation into their PAR cycles. However, challenges were noted in application of some of the tools for reflection with partners, such as use of diaries and e‐diaries, which were an added duty for them. Reflection in routine meetings with partners, across partners (e.g., across sites) and in standard fora was more feasible, though still demanding in terms of time for participants. In most programs, researchers took responsibility for eliciting, synthesizing, and reporting the reflexive elements and working to enact the adaptations deliberated over and agreed through the consensus building processes.

For monitoring and learning, as interactions are frequent and continuous, there is a challenge involved in maintaining good records and managing routine documentation. The routine documentation of events, interactions, meetings, research activities, and outputs make up a huge resource of information and analyzing it, using the right information in the right place, needs careful planning from the start. A number of the programs developed regular (e.g., monthly) reflection and planning meetings with researchers and local partners to record and reflect on joint activities. Being systematic and transparent in analysis and sense‐checking across teams and with partners is crucial. Use of virtual platforms and spaces and shared repositories for program data have also been supportive, especially during times of crisis such as COVID.

Another challenge lies in developing messages which have resonance beyond the specific sites, which requires that wide‐ranging participation is engaged to ensure that lessons are developed which can inform a broader geography and are shared in a range of formats beyond the traditional incentivized “products” from research (papers and conference presentations). VAPAR, for example, developed a radio series on CHW roles and functions in TsSonga, research briefs in English and local languages, podcasts, YouTube content, and blogs.^17^

#### 3.3.2 Demonstrating impact

It is complex to ascertain and demonstrate impact from learning sites, given the complexity of the environment, the intrinsic as well instrumental role that they can play, and the dynamic engagement over time. However, this is important, including to funders. All programs used end‐of‐cycle evaluations using participatory methods. One response was to develop a theory‐based evaluation of the platform (e.g., for VAPAR), which allowed the different domains to be tracked to assess the contribution of the program.^18^ While health effects are important, the significance of building “intermediate” domains, such as trust, capacity, confidence, and systemic relationships is highlighted in these evaluations, tracked largely qualitatively through interviews and process documentation. These have benefits beyond the immediate period and (potentially) constitute real system strengthening.^19^

## 4 GAINS ACHIEVED

Gains documented by the programs included important domains such as giving voice to communities, increasing skills and confidence of managers, and creating and sustain spaces and processes to support cadres that link these two groups, such as CHWs. Increased researcher skills and increased demand for local evidence have also been identified, along with improved relationships between different groups, with learning sites often providing much‐needed spaces for collaboration. These in turn can trigger innovations, improvements in service funding and delivery, and changes to policy and practice.

### 4.1 Giving voice to communities and connecting them to service providers

CEGSS has done several participatory impact assessments in which organized users define their own impacts. In these exercises, impacts included increased knowledge and capacity to navigate the public system and learning about methods and tools to monitor public policies and services.^20^, ^21^ Equally, VAPAR has documented community stakeholders realizing and developing shared capacities to use their voice, while service providers found that the learning platform was a safe way to engage with users in spaces which had hitherto been lacking, with discontent frequently expressed in violent protest given the absence of constructive engagement channels. The process shifted otherwise disconnected actors toward more constructive dialogue and collective mindsets.^4^

### 4.2 Increasing skills and confidence among managers

Programs focused on managers (e.g., PERFORM2Scale) noted improved confidence and independence in problem‐solving and strategy development in this group. Participants also reported more innovative and creative thinking (in part because of the lack of additional resources, which necessitated this), as well as more regular meetings of the management groups. In ReSYST, an evaluation of a multifaceted leadership development program for mid‐level managers and facility managers embedded within the learning site in Kenya was undertaken.^22^ It found that managers reported greater recognition of the importance of health system software (values, belief systems, and relationships) and that the training also created spaces for managers to share experiences, reflect upon, and nurture social competences.

### 4.3 Empowering linking cadres such as CHWs

As a cadre connecting health systems with communities, CHWs have been engaged and played central roles, in learning sites. For example, in VAPAR and at the onset of the COVID‐19 pandemic, the process was co‐re‐designed to focus to CHWs' roles, functions, and relationships with both communities and the health system. In the context of suboptimal integration of CHWs and poor working conditions, an evaluation of the VAPAR engagement of CHWs found improvements to key CHW capabilities in community mobilization as well as new skills and confidence in complex analysis, public speaking, and reporting, alongside greater role clarity. In this evaluation, CHWs reported a “triple benefit”: strengthened relationships with communities, better peer‐to‐peer relationships and support, and improved recognition by the health system.^6^

### 4.4 Benefits for researchers and increasing demand for local data

All programs note the benefits for researchers of engaging in learning sites, including gaining a richer understanding of resources and challenges in the local health system, but also deepening relationships with policy‐makers (beneficial for research and also its uptake) and identifying opportunities and priorities for future research (as highlighted, e.g., in^5^).

In addition, in many settings, health data are fed upward but not used locally for priority setting and resource allocation, which in decentralized contexts is particularly problematic. Learning sites encourage more active use of co‐developed, relevant, and owned local data (in its many forms, including community and managerial perceptions, as well as surveys and routine sources). In ReBUILD, for example, appreciation of support in analyzing local data was noted from the municipality, which has become a champion in this area and has started to allocate funds for research in annual plans.^7^

### 4.5 Building local relationships and intersectoral work

Spaces for collaborative reflection, dialogue, and planning for action across organizational silos and district teams (and between system and community) are rare in most settings, and where they exist can be poorly functional,^23^ and this was a key feature that the learning sites aimed to address. Once established and experienced, this was typically highly appreciated by participants, who were able to develop new relationships and gain increased appreciation of structures, opportunities, and responsibilities for collective action.^24^

A number of the programs engaged with a wide variety of sectors and local actors (beyond health) and documented strengthened relationships and teamwork between them. For example, in VAPAR, work on the issues of access to water, drugs, and alcohol mobilized partnerships across sectors, a feature that was noted as beneficial in working efficiently and avoiding duplication by those involved.^8^ The focus on the municipality in ReBUILD improved coordination across wards and local leaders, with potential benefits beyond the health sector. This included sensitizing the non‐health stakeholders in the municipality – mainly the ward level officials who are the key decision makers – on health and health systems, which are a new area of responsibility for them in the decentralized system. Different sectoral sections within the municipality also started holding regular coordination meetings – the only platform where all sectors come together to discuss progress, challenges, and explore opportunities for collaboration and integration.

In Guatemala, as the network of Community Defenders acquired more experience and knowledge, they became more effective in monitoring services, mediating and solving conflicts (when there are complaints between providers and users of services), and helping health providers and authorities in communicating key messages and priorities to communities. As a result, their recognition by providers and authorities grew, leading to demand for them to support other programs such as nutrition, environmental health and school health. The increase in recognition and demand also contributes to sustaining the work.

### 4.6 Supporting tailored innovations to strengthen systems

All of the programs noted gains in relation to their core ambition to create conditions for dialogue and innovation to strengthen local systems. For example, ReSYST has documented the co‐production of local interventions—small wins that increase the confidence and agency of managers to make a difference in their sphere of influence and can have ripple effects to other areas of engagement. In Kenya, the platform brought together staff across levels of the now‐fragmented decentralized health system to share concerns and perspectives, resulting in practical improvements such as new TORs for health facility in‐charges and new induction plans. In PERFORM2Scale, health workforce and service delivery problems were addressed, including implementing changes to areas such as induction, attendance, supervision, performance appraisal, and rewards and sanctions. In ReBUILD, the co‐created action plans were owned by the local government and the actions (immediate ones) were incorporated into the municipality annual workplan and budget, while agreeing to gradually incorporate other actions in longer‐term plans and practice. These measures address strengthening governance mechanisms at municipality and health facilities and improving planning using local evidence. These gains were achieved through the strategies highlighted above, including continuous reflective engagement, respectful partnership, creating safe spaces, and capacity development.

### 4.7 Improvements in service funding and delivery

The advocacy of organized users of services has resulted in improved funding for local healthcare services in Guatemala. Still, many bottlenecks are caused by actions at central levels, and therefore, CEGSS is supporting organized users to also engage at national level.

In other sites, there is evidence of improved service delivery linked to learning site activities. For example, improvements in access to services were shown in a district in Ghana which selected the problem of low outpatient department (OPD) attendance (PERFORM2Scale). By strengthening Community Health Committee meetings and the regular engagement of health staff with the community, outpatient attendance increased, as well as community participation in health campaigns, such as mass drug administration for neglected tropical diseases. These improvements were recognized in the annual district review and resulted in that district being awarded “best performing district in the region.”^9^ Improvements in patient adherence to HIV/AIDS treatment and better patient tracking were among the benefits documented for VAPAR.^24^

### 4.8 Contributing to broader policy and practice

In addition to local benefits, all learning sites aim to develop lessons that can be shared horizontally (e.g., to other municipalities or districts) but also vertically, in informing national policy and practice. This is often done through engagement of participants in technical working groups at different levels. For example, in Kenya, the ReSYST learning site contributed to the Kilifi County Health Facilities Improvement Fund Bill in 2016 and the Ministry of Health Guidelines for County Level Health Sector Annual Work Planning and Performance Review Processes in 2018. There has also been considerable effort put into scaling up and sharing lessons more widely—for example, VAPAR has seeded a new learning platform in a neighboring province, and is now responding to demand from the provincial health authority to implement the CHW training across Mpumalanga. In PERFORM2Scale, scale‐up strategies were developed in each country with integration of the management strengthening intervention into policies and routine practice in Uganda and Malawi, all in the absence of additional financial support.

## 5 DISCUSSION

Reflecting across the five programs, which incorporate a number of sites, it is clear that the learning site approach can be potentially very powerful in developing relationships, capacities, and local innovations. The aim is to break down the traditional relationship between researcher and researched, with researchers working with local actors to co‐create and study participatory solutions. The risks of this are well recognized, in the form of partiality and challenges of generalizability and scale‐up, as the engagement needs to be embedded, intensive and sustained. However, if health policy and systems research aims at impact, then this approach is worth investing in, as the process of research is itself the means through which capacity can be built and change achieved, breaking down the barrier commonly found between research and research uptake and empowering local actors (addressing problematic and exploitative norms of extractive and decontextualize working that some research has adopted in the past).

There has been a focus on the importance of fostering “learning health systems” in low‐ and middle‐income countries in recent years, with a review finding that learning comes from the connection between information, deliberation, and action, which can be fostered by creating spaces and resources for communities, staff, and managers to share experiential knowledge.^25^ This is exactly the role which learning sites have been trying to take on, and it remains crucial, particularly when systems face resource constraints and everyday or acute stressors and shocks.

The programs, which are only a small subset of existing learning sites but which do include a wide range of geographies and focal areas, highlight some important contextual factors which support effective learning sites, including pre‐existing relationships and infrastructure (such as the HDSS platform in Mpumalanga and prior programs for ReBUILD, PERFORM2Scale, and ReSYST), along with supportive organizational environments, local champions, and stable, longer‐term funding.^26^ Within the sites, regularity of engagement over time (e.g., over repeated PAR cycles), building respectful relations, fostering capabilities and mutual connections, adaptability, and linking participants to relevant external resources (including peer‐to‐peer learning) are highlighted. Attention to power relations within the group is also key, although there can be tensions to be carefully managed here, including between giving voice to marginalized groups but also ensuring that those with power to act on problem areas are involved. Engaging wider actors at local and higher system level is also required, as constraints are generally driven by multi‐level factors that local actors alone cannot address.

It is clear that the role of learning site facilitation is highly skilled, and includes the ability to build relationships with and across partners, create constructive and respectful engagement, maintain group cohesion and enthusiasm, find opportunities to input to local plans without over‐committing, be responsive to local requests, communicate effectively to multiple actors, be reflective about progress and lessons, and be able to document systematically and share findings widely. These roles can be shared across the team and partners and can be taught, nurtured, and supported, where not inherent.

Sustainability is partly achieved through the enhanced capabilities and connections highlighted above, but in addition, the learning sites undertake deliberate efforts to encourage incorporation over time in routine local processes, which can support learning activities beyond the funding timetable of research programs. This is challenging, but crucial as learning sites require long‐term engagement. Evidence of demand for continuation of the platforms at local level in these programs, such as the scale‐up of the VAPAR work to provincial level and the growth and long timespan of the CEGSS program, is encouraging in that respect.

## CONFLICT OF INTEREST STATEMENT

The authors have no conflict of interest to declare.
